# LIVING DONOR LIVER TRANSPLANT FOR INTRAHEPATIC CHOLANGIOCARCINOMA. AN INITIAL BRAZILIAN EXPERIENCE

**DOI:** 10.1590/0102-6720202400045e1839

**Published:** 2024-12-02

**Authors:** Eduardo de Souza Martins FERNANDES, Felipe Pedreira Tavares de MELLO, Ronaldo de Oliveira ANDRADE, Camila Liberato GIRÃO, Camila CESAR, Leandro Savattone PIMENTEL, Henrique Sergio Moraes COELHO, Samanta Teixeira BASTO, Munique SIQUEIRA, Anderson BRITO, Claudia Cristina TAVARES DE SOUSA, Tercio GENZINI, Orlando Jorge Martins TORRES

**Affiliations:** 1 São Lucas Hospital, Departament of Gastrointestinal and Liver Transplant Surgery – Rio de Janeiro (RJ), Brazil; 2 São Lucas Hospital, Department of Gastroenterology and Hepatology – Rio de Janeiro (RJ), Brazil; 3 Universidade Federal do Maranhão, Hepatopancreatobiliary and Liver Transplant Unit, Department of Gastrointestinal Surgery – São Luis (MA), Brazil.

**Keywords:** Cholangiocarcinoma, Liver transplantation, Tissue donors, Drug therapy, Colangiocarcinoma, Transplante de fígado, Doadores de tecidos, Tratamento farmacológico

## Abstract

**BACKGROUND:**

Intrahepatic cholangiocarcinoma (iCCA) was considered a contraindication for liver transplantation. However, recent studies have shown that highly selected cases of patients with a good response to neoadjuvant therapy may achieve acceptable survival rates when following liver transplantation.

**AIMS:**

To present two cases of patients with iCCA, without extrahepatic disease, who underwent living donor liver transplantation after receiving neoadjuvant chemotherapy.

**METHODS:**

Two cases of patients with histopathological diagnosis of locally advanced iCCA, ineligible for resection and without evidence of extrahepatic disease, are presented.

**RESULTS:**

These patients underwent at least nine sessions of neoadjuvant chemotherapy, including Gemcitabine and Cisplatin, with or without the addition of immunobiological agents, resulting in a radiological tumor response. They subsequently underwent living donor liver transplantation. The average follow-up time was 15 months, with no clinical or radiological signs of disease.

**CONCLUSIONS:**

In well-selected patients without extrahepatic disease, living donor liver transplantation represents a potential therapeutic option for iCCA.

## RESUMO

**RACIONAL:**

O colangiocarcinoma intra-hepático (CCi) foi considerado uma contraindicação para transplante hepático. No entanto, estudos recentes têm demonstrado que casos altamente selecionados de pacientes com boa resposta à terapia neoadjuvante podem ter uma taxa de sobrevida aceitável ao serem submetidos a transplante hepático.

**OBJETIVOS:**

Apresentar dois casos de pacientes com colangiocarcinoma intra-hepático sem doença extra-hepática submetidos a transplante hepático de doador vivo, após quimioterapia neoadjuvante.

**MÉTODOS:**

Dois pacientes com diagnóstico histopatológico de Cci, localmente avançado, inelegíveis para ressecção e sem evidência de doença extra-hepática.

**RESULTADOS:**

Esses pacientes foram submetidos a pelo menos nove sessões de quimioterapia neoadjuvante incluindo Gemcitabina e Cisplatina com ou sem adição de agentes imunobiológicos e com resposta tumoral radiológica. Em seguida, foram submetidos a transplante hepático de doador vivo. O tempo médio de acompanhamento foi de 15 meses, sem sinais clínicos ou radiológicos de doença.

**CONCLUSÕES:**

Em pacientes bem selecionados, sem doença extra-hepática, o transplante de fígado de doador vivo é uma opção terapêutica potencial para colangiocarcinoma intra-hepático.

## INTRODUCTION

Intrahepatic cholangiocarcinoma (iCCA) is the second most common type of liver cancer. Its incidence and mortality rates have been increasing and are projected to become a greater burden in the future, with poor long-term survival^17^.

Current evidence suggests that curative-intent treatment is limited to liver resection^10,17^. However, only 30-40% of patients are considered resectable at diagnosis. This is partially explained by late diagnosis and locally advanced disease. An important proportion of patients with early iCCA might not be fit for surgery due to chronic liver disease and poor functional reserve^1,3^.

The recent uprise of transplant oncology has led to an increased use of liver transplantation (LT) for solid tumor treatment^5^. This trend is well depicted by the experience in perihilar cholangiocarcinoma, where better selection protocols were crucial to achieving acceptable outcomes^4^. Recent studies have demonstrated that, under strict protocols, LT can achieve impressive outcomes, with 65-70% 5-year survival rates^12,14,15,18^. Thus, there is a strong rationale for proposing liver transplantation for iCCA^5^. Liver transplant has been investigated by various centers in well-selected patients, particularly in 2 scenarios: early lesions smaller than 2 cm in patients with chronic liver disease, and locally advanced tumors with a good response to neoadjuvant treatment^2,6,9,11,13,16^. This study aimed to present two cases of living donor liver transplantation for intrahepatic cholangiocarcinoma and discuss the potential benefits of this treatment strategy.

## CASE REPORT AND TECHNIQUE

**Figure 1:**
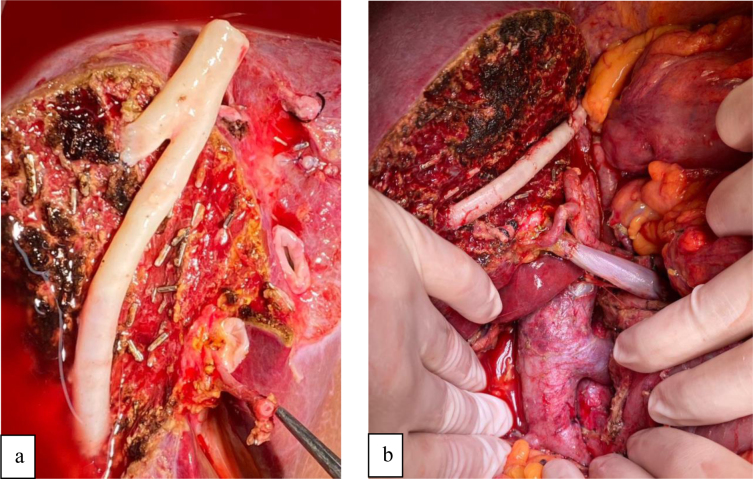
a) Right lobe graft donated by her son. b) Venous reconstruction of V5 and V8 using a cadaveric iliac artery graft during the back table procedure.

Case 1: Female, 66 years old, body mass index (BMI) of 23, with a history of hypertension and *diabetes mellitus*. Routine abdominal ultrasound revealed hepatic nodules. During the diagnostic workup, the patient developed ascites and an increase in the number or size of the lesions. A liver lesion biopsy was performed under interventional radiology guidance, and the histopathological analysis confirmed well-differentiated iCCA. Staging radiological examinations were conducted, including positron emission tomography-computed tomography scan (PET-CT), showed no evidence of extrahepatic disease, while magnetic resonance imaging (MRI) revealed a suspicious lymph node in the hepatogastric ligament. Neoadjuvant chemotherapy with Gemcitabine and Cisplatin was initiated. The patient completed a total of 15 cycles and achieved lesion reduction. No extrahepatic disease was found, and laparoscopy with intraoperative frozen section biopsy of the suspicious lymph node showed no evidence of malignancy. However, macroscopic cirrhotic changes were observed in the liver, despite negative serologies. Esophagogastroduodenoscopy revealed esophageal varices, and Doppler ultrasound diagnosed portal vein thrombosis (Table 1). The case was referred for evaluation by the Transplant Technical Chamber and approved for living donor liver transplantation. During exploratory laparotomy, the recipient underwent a thorough inspection for extrahepatic disease. The patient received a right lobe graft donated by her son. Venous reconstruction of V5 and V8 was performed using a cadaveric iliac artery graft during the backtable procedure (Figure 1a and b). Total hepatectomy was performed, along with lymphadenectomy of stations 7, 8, 9, 10, 12, 13, 16a2, 16b1, and 17. Due to portal vein thrombosis, open mechanical thrombectomy and temporary portocaval anastomosis were carried out until graft retrieval from the donor. Implantation was performed using the piggyback technique, and biliary reconstruction was accomplished through bilioenteric Roux-en-Y anastomosis. The histopathological analysis of the specimen revealed cholangiocarcinoma metastasis in 2 of 7 lymph nodes in the hepatic artery, along with lymphovascular and perineural invasion. Postoperatively, the patient presented metabolic ileus on the tenth day, which resolved spontaneously. The patient was discharged on the twentieth day without further complications. Quarterly laboratory and radiological evaluations were conducted for follow-up purposes. The patient has now completed 23 months without recurrence.

**Table 1.** Case presentation.

.	Recipient 2	Recipient 1
Radiographic (pre-transplant)		
Stage	T2aN0M0	T2bN+M0
Number of lesions	1	2
Maximum size of lesions (cm)	6.5 x 4cm	7.9cm
Explant		
Stage	T2bN0M0	T2N1M0
Number of Lesions (cm)	4	1
Maximum size of lesions (cm)	5.5	12
Location	Bilobar	Bilobar
Differentiation	Moderate to well	Moderate
Lymphovascular invasion	No	Yes
Perineural invasion	No	Yes
Necrosis (%)	35%	No
**Recipient demographics**	Recipient 1	Recipient 2
Age	54y	63y
BMI	23	23
Sex	Female	Female
Diabetes	No	Yes
Hypertension	No	Yes
Cirrhosis	Yes – HCV	No
Smoking	No	No
MELD at transplant	7	7
Duration from Diagnosis (months)	6	12
Neoadjuvant Chemotherapy	Gemcitabine + Cisplatin	Gemcitabine + Cisplatin
Immunotherapy	Durvalumab	No
Neoadjuvant cycles	9	15
Operative characteristics		
Venous reconstruction	Yes (Dominant V8 + IVC – PTFE)	Yes (V8 and V5 – iliac graft)

T: tumor staging; N: lymph nodes; M: metastasis; y: years; BMI: body mass index; HCV: hepatitis C virus; MELD: model for end-stage liver disease; V: vein; IVC: inferior vena cava; PTFE: polytetrafluoroethylene.

**Figure 2:**
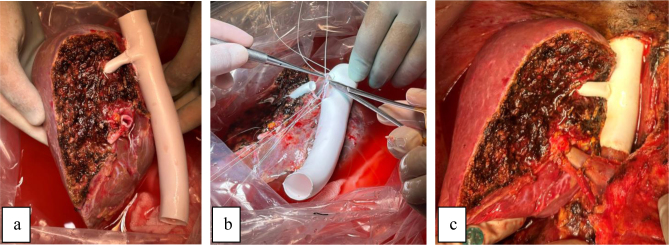
a) On the back table, after perfusing the right lobe graft, venous reconstruction was performed for the dominant drainage of segment VIII using a PTFE graft; b) neohepatic vena cava; and c) the graft reperfusion occurred uneventfully, followed by arterial and biliary anastomosis without complications.

Case 2: Female, 54 years old, BMI of 23, previously treated for hepatitis C with Metavir. The patient presented a single lesion involving segments I and VIII, in close contact with the inferior vena cava, with no evidence of extrahepatic disease. A diagnosis of cholangiocarcinoma was confirmed through a percutaneous biopsy performed by interventional radiology. Initially, surgical treatment was proposed, and during the procedure, a cirrhotic liver and two satellite lesions in segments III and VIII were identified and confirmed by frozen section examination. No signs of extrahepatic disease were identified, and perihilar hepatic lymph nodes were biopsied, showing no evidence of malignancy on frozen section examination. The patient was then referred for neoadjuvant chemotherapy and immunotherapy with Gemcitabine, Cisplatin, and Durvalumab. After 6 months and 9 cycles of chemotherapy, the disease remained stable, and the patient was approved by the Transplant Technical Chamber for living donor liver transplantation. Total hepatectomy was performed using the cross-clamp technique due to the lesion’s proximity to the retrohepatic vena cava. On the back table, after perfusing the right lobe graft, venous reconstruction was performed for the dominant drainage of segment VIII using a polytetrafluoroethylene (PTFE) graft (neohepatic vena cava), and the graft reperfusion (Figure 2) occurred uneventfully, followed by arterial and biliary anastomosis without complications. Histopathological analysis of the specimen showed clear margins, absence of lymph node metastasis, and no lymphovascular or perineural invasion. There were no postoperative complications, with a hospital stay of 11 days. A liquid biopsy performed four weeks later showed no evidence of malignancy. Radiological and laboratory evaluations after six months revealed no signs of recurrence.

## DISCUSSION

iCCA is a common liver tumor, and liver resection, particularly major hepatectomy, is the treatment with curative intent. Five-year overall survival after resection has been reported to range from 20 to 42%, while tumor recurrence in two years is approximately 56%^7^. The future liver remnant is the primary factor for resectability, in addition to vascular resection and reconstruction. For unresectable tumors, systemic therapy remains the palliative modality, without long-term survival. Transplant oncology is a new concept designed to optimize the treatment of patients with hepatobiliary cancers^4^. Liver transplantation is part of this concept, extending the limits of conventional resection and incorporating clinical and immunologic anticancer strategies, including transplantation and hepatobiliary surgeons, immunologists, medical and radiation oncologists, hepatologists, and gastroenterologists to maximize outcomes and aim for a cure^5,7,14,15^.

In the present cases, both patients underwent neoadjuvant chemotherapy cycles with Gemcitabine and Cisplatin. In one of the cases, immunotherapy cycles with Durvalumab were also administered. Both patients achieved either radiological response or tumor stability for a minimum of 6 months, subsequently undergoing total hepatectomy with extensive lymphadenectomy (stations: 7, 8, 9, 10, 12, 13, 16a2, 16b1, 17), followed by living donor liver transplantation (right lobe). Although the number of patients is limited and the follow-up period relatively short, this report aimed to address a highly relevant and current topic in the treatment of iCCA^7^. Major European and American centers have reported promising five-year survival outcomes for patients with unresectable iCCA and liver-confined disease who underwent neoadjuvant chemotherapy followed by deceased donor liver transplantation^2,6,9,11,13^. However, in Brazil, the use of deceased donor grafts is not authorized, even after careful patient selection, and is only possible with living donors.

Considerable debate revolves around the significance of lymphadenectomy during iCCA surgery, as lymph node metastasis is present in 16 to 45% of cases and represents a major prognostic factor^8,16^. In this context, extended lymphadenectomy performed during the surgical procedure, although not clearly associated with local recurrence, has been directly linked to improved survival outcomes without significant morbidity in non-cirrhotic patients. Therefore, further studies are still warranted to evaluate the role of extended lymphadenectomy in liver transplantation for iCCA treatment.

## CONCLUSION

In well-selected patients without extrahepatic disease and with a good response to neoadjuvant therapy, liver transplantation appears to be a potential therapeutic option for intrahepatic cholangiocarcinoma. Living donor liver transplantation should be indicated after careful patient selection if deceased donor grafts are not permitted. More data is needed to refine selection criteria and perioperative management.
